# Supplementary material for: Splice-Junction-Based Mapping of Alternative Isoforms in the Human Proteome
Source: Cell Rep. Author manuscript; Available in PMC 2020 Jan 15. (PMC6961840; doi:10.1016/j.celrep.2019.11.026)
Supplement: 2 [file NIHMS1546469-supplement-2.zip › DF1/PXD006675/VentricularSeptum/VentricularSeptum_25_TPM1_AISEELDHALNDMTSGP.pdf]

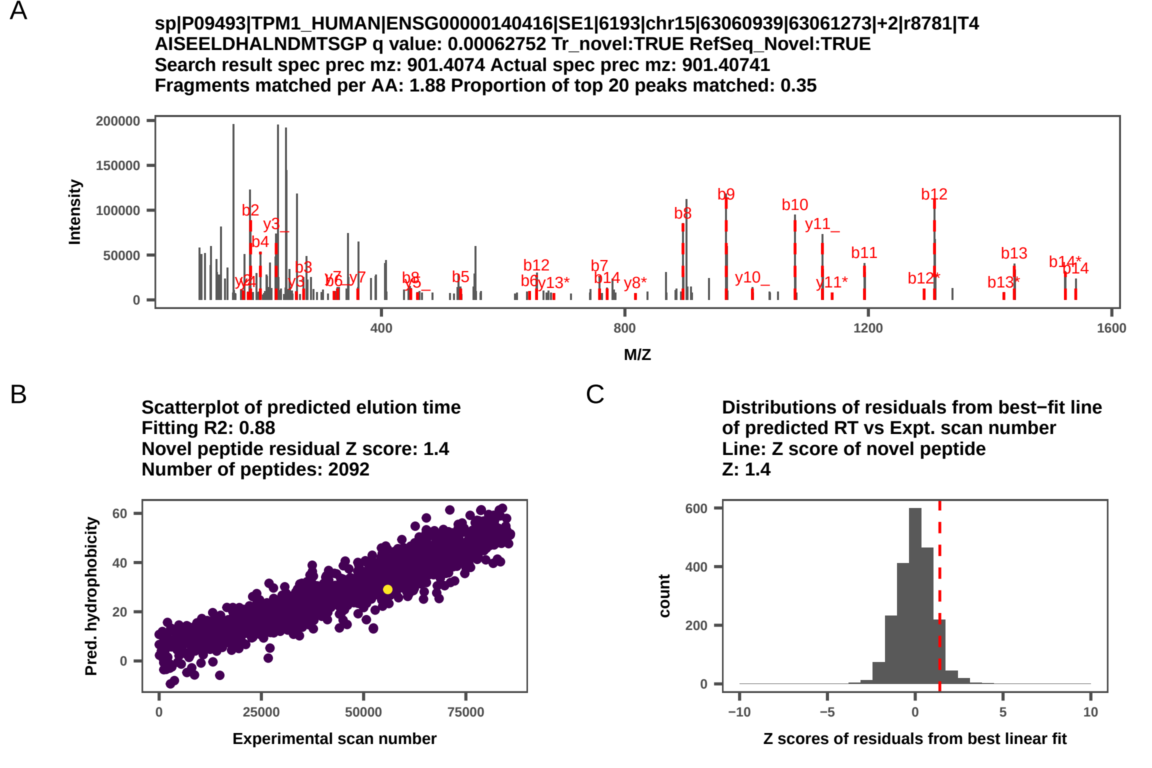
A
sp|P09493|TPM1_HUMAN|ENSG00000140416|SE1|6193|chr15|63060939|63061273|+2|r8781|T4
AISEELDHALNDMTSGP q value: 0.00062752 Tr_novel:TRUE RefSeq_Novel:TRUE
Search result spec prec mz: 901.4074 Actual spec prec mz: 901.40741
Fragments matched per AA: 1.88 Proportion of top 20 peaks matched: 0.35
Intensity
200000
150000
100000
50000
0
400
800
1200
1600
M/Z
b2
y3_
b4
b3
y2
y3
b6_
y7
b8
y5_
b5
b12
b6
y13*
b7
b14
y8*
b8
b9
y10_
b10
y11_
y11*
b11
b12
b12*
b13
b13*
b14*
b14
B
Scatterplot of predicted elution time
Fitting R2: 0.88
Novel peptide residual Z score: 1.4
Number of peptides: 2092
Pred. hydrophobicity
60
40
20
0
0
25000
50000
75000
Experimental scan number
C
Distributions of residuals from best−fit line
of predicted RT vs Expt. scan number
Line: Z score of novel peptide
Z: 1.4
count
600
400
200
0
−10
−5
0
5
10
Z scores of residuals from best linear fit
